# Genomic analysis of severe COVID-19 considering or not asthma comorbidity: GWAS insights from the BQC19 cohort

**DOI:** 10.1186/s12864-024-10342-x

**Published:** 2024-05-16

**Authors:** Omayma Amri, Anne-Marie Madore, Anne-Marie Boucher-Lafleur, Catherine Laprise

**Affiliations:** 1 Centre intersectoriel en santé durable, Université du Québec à Chicoutimi, Saguenay, Québec, G7H 2B1 Canada; 2 Département des sciences fondamentales, Université du Québec à Chicoutimi, Saguenay, Québec, G7H 2B1 Canada; 3 Centre de recherche du Centre intégré universitaire de santé et de services sociaux du Saguenay–Lac-Saint-Jean, Saguenay, Québec, G7H 7K9 Canada

**Keywords:** COVID-19, Severe COVID-19, Asthma, GWAS, BQC19

## Abstract

**Background:**

The severity of COVID-19 is influenced by various factors including the presence of respiratory diseases. Studies have indicated a potential relationship between asthma and COVID-19 severity.

**Objective:**

This study aimed to conduct a genome-wide association study (GWAS) to identify genetic and clinical variants associated with the severity of COVID-19, both among patients with and without asthma.

**Methods:**

We analyzed data from 2131 samples sourced from the *Biobanque québécoise de la COVID-19* (BQC19), with 1499 samples from patients who tested positive for COVID-19. Among these, 1110 exhibited mild-to-moderate symptoms, 389 had severe symptoms, and 58 had asthma. We conducted a comparative analysis of clinical data from individuals in these three groups and GWAS using a logistic regression model. Phenotypic data analysis resulted in the refined covariates integrated into logistic models for genetic studies.

**Results:**

Considering a significance threshold of 1 × 10^−6^, seven genetic variants were associated with severe COVID-19. These variants were located proximal to five genes: sodium voltage-gated channel alpha subunit 1 (*SCN10A)*, desmoplakin (*DSP)*, RP1 axonemal microtubule associated (*RP1*), IGF like family member 1 (*IGFL1*), and docking protein 5 (*DOK5*). The GWAS comparing individuals with severe COVID-19 with asthma to those without asthma revealed four genetic variants in transmembrane protein with EGF like and two follistatin like domains 2 (*TMEFF2*) and huntingtin interacting protein-1 (*HIP1*) genes.

**Conclusion:**

This study provides significant insights into the genetic profiles of patients with severe forms of the disease, whether accompanied by asthma or not. These findings enhance our comprehension of the genetic factors that affect COVID-19 severity.

**Key messages:**

- Seven genetic variants were associated with the severe form of COVID-19;
- Four genetic variants were associated with the severe form of COVID-19 in individuals with comorbid asthma;
- These findings help define the genetic component of the severe form of COVID-19 in relation to asthma as a comorbidity.

## Introduction

In March 2020, the World Health Organization (WHO) officially declared the outbreak of coronavirus disease 2019 (COVID-19), caused by infection with severe acute respiratory syndrome coronavirus 2 (SARS-CoV-2), as a global pandemic [1]. By May 2023, this disease engendered a staggering 796 million infections worldwide, resulting in approximately 6.9 million deaths, equating to a mortality rate of 0.9% [2]. The range of COVID-19 symptoms varies from asymptomatic to fatality in severe cases. The majority of those infected with the virus experience mild symptoms such as cough, fever, headache, asthenia, anosmia, and ageusia [3]. However, certain cases require hospitalization and mechanical ventilation to prevent severe respiratory failure [4]. Hospitalized patients, advanced age, male sex [5, 6], and underlying medical conditions such as hypertension, obesity, and diabetes, exhibit strong correlations with mortality [5–9]. The severity of COVID-19 may be affected by other factors, such as autoimmune diseases and genetic variations, which either enhance the susceptibility to severe outcomes or protect against them [10].

Various studies have work elucidating the genetic mechanisms that influence the severity of COVID-19 and associated different loci as 3p21.31 and 9q34.2 to respiratory failure and severe complications [11–13]. Concerning the 9q34.2 locus, it harbors the ABO, alpha 1-3-N-acetylgalactosaminyltransferase and alpha 1-3-galactosyltransferase (*ABO*) gene, which may modulate COVID-19 susceptibility and symptom severity through immunological interactions and inflammatory responses [11, 14]. Earlier research has posited that the *ABO* gene, jointly associated with asthma and severe COVID-19, may partly explain the association between these conditions [15]. Additionally, studies have reported a lower prevalence of asthma among COVID-19 patients compared to the general population [16, 17], suggesting potential resistance conferred by asthma against viral infection [18]. Furthermore, it has been proposed that allergic asthma may enhance immunity by inducing eosinophilia and a type 2 helper T cell (Th2) inflammatory response via the interleukin (IL)-13 pathway [19]. Further genetic investigations have suggested the involvement of the 12q24.13 locus, encoding oligo-adenylate synthetases (*OAS*) family, in asthma’s protective mechanisms via airway remodeling [20] and in COVID-19 [21], through mechanisms aiding in viral ribonucleic acids (RNAs) degradation and viral replication inhibition by activating latent ribonuclease [22].

The objective of this study is, firstly, to identify a genetic profile distinguishing patients with severe COVID-19 from those experiencing mild-to-moderate manifestations within the Quebecois population, and secondly, to establish a genetic profile for severe COVID-19 patients afflicted with asthma compared to those without asthma.

## Methods

**Fig. 1:**
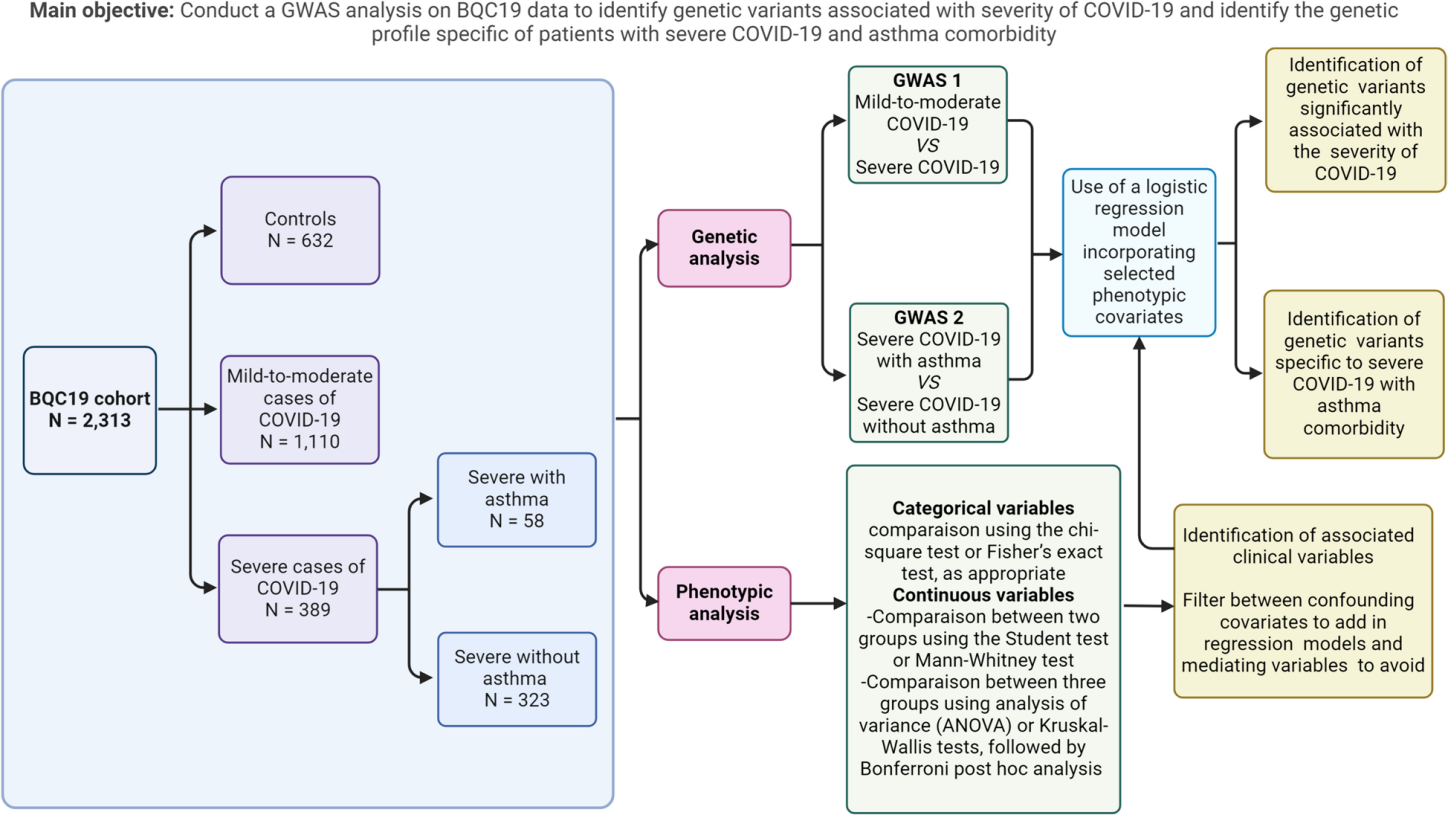
Explanatory diagram of the study design and objectives (figure created with BioRender.com)

A schematic view of the study design is presented in Fig. 1, with a brief description of the study population and the analyses performed, including the main objectives.

### Study population

The study participants were sourced from the *Biobanque québécoise de la COVID-19* (BQC19) (https://www.quebeccovidbiobank.ca/) established in Quebec, Canada. The primary objective of this biobank is to enable the scientists to access biological materials and data to facilitate COVID-19 research. Ethical approval was granted by the Research Ethics Board of the *Centre intégré universitaire de santé et de services sociaux du Saguenay–Lac-Saint-Jean* (IDs: 2022–388, 2021–026). Informed consent was obtained from all participants or their legal guardians in cases where the individual was unable to provide consent or was below 18 years of age [23].

**Fig. 2:**
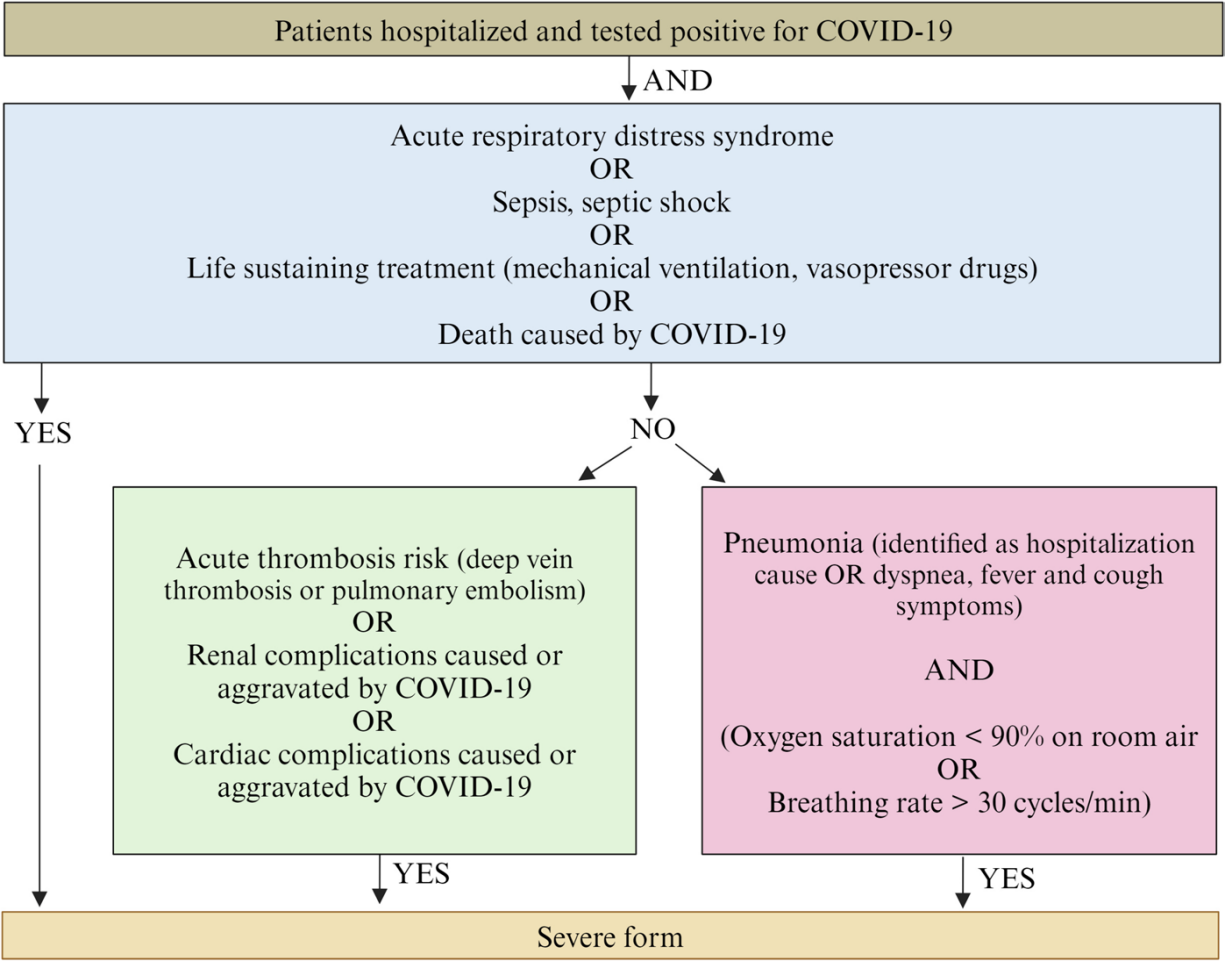
Flowchart for COVID-19 severity criteria. Participants are categorized as experiencing a severe stage of COVID-19 based on two primary criteria: a positive COVID-19 test and the necessity for hospitalization. Additionally, in conjunction with these two criteria, participants were required to satisfy the specified conditions outlined in one of the three other sections to be considered as having a severe manifestation. The figure is generated using BioRender.com

This study involved 2131 patients aged from 2 months to 102.7 years old (Table 1). The samples and clinical data were sourced from both non-hospitalized and hospitalized individuals. All participants agreed to participate in the local clinical COVID-19 testing using SARS-CoV-2 RNA reverse transcriptase polymerase chain reaction (RT-PCR). Among these participants, 1499 tested positive for SARS-CoV-2, whereas 632 tested negative. SARS-CoV-2 PCR-negative patients were recruited as controls while patients with COVID-19 were categorized into two groups based on severity: 389 severe cases and 1110 mild-to-moderate cases. The severity of COVID-19 was classified based on WHO established criteria (Fig. 2) [24].

**Table 1:** Summary of the studied population’s traits

		Patients with COVID-19 (*n* = 1499)					
					Severe with known asthma status (*n* = 381)		
	Controls (*n* = 632)	Mild-to-moderate (*n* = 1110)	Severe (*n* = 389)	*P* value^f^	Without asthma (*n* = 323)	With asthma (*n* = 58)	*P* value^g^
**Sex, M:F**	1.00:0.97 ^a^	*1.00:1.18* ^*a*^	*1.00:0.59* ^*b*^	< 1.000 × 10^−6^	1.00:0.55 ^a^	1.00:0.87 ^a^	0.140
**Age, mean (range)**	*60.80 (0.20–101.50)* ^*a*^	*58.82 (0.20–102.70)* ^*b*^	*63.93 (0.70–97.70)* ^*a*^	0.103 × 10^−4^	64.78 (0.70–97.70) ^a^	60.32 (32.50–94.40) ^a^	0.050
**Age, median**	*63.25* ^*a*^	*59.20* ^*b*^	*66.20* ^*a*^		66.60 ^a^	57.25 ^a^	
**BMI (kg/m**^**2**^**), mean (SD)**	*26.77 (6.17)* ^*a*^	*27.83 (6.44)* ^*b*^	*28.89 (7.05)* ^*c*^	0.122 × 10^−3^	*28.17 (6.37)* ^*a*^	*33.70 (8.49)* ^*b*^	0.519 × 10^−3^
**Hospitalization, n (%)**	*575 (91)* ^*a*^	*759 (68)* ^*b*^	*389 (100)* ^*c*^	< 1.000 × 10^−6^	323 (100) ^a^	58 (100) ^a^	1.000
**Dyspnea, n (%)**^**d**^	*282 (71)* ^*a*^	*518 (63)* ^*b*^	*323 (90)* ^*c*^	< 1.000 × 10^−6^	270 (90) ^a^	49 (88) ^a^	0.477
**Systemic corticosteroids, n (%)**^**d**^	47 (8) ^a^	72 (7) ^a^	30 (8) ^a^	0.861	24 (8) ^a^	5 (9) ^a^	0.787
**ACE inhibitors, n (%)**^**d**^	158 (26) ^a^	*231 (23)* ^*a*^	*140 (37)* ^*b*^	< 1.000 × 10^−6^	115 (36) ^a^	25 (44) ^a^	0.298
**Respiratory rate (breaths per minute), mean (SD)**	*20.97 (7.06)* ^*a*^	*22.64 (5.81)* ^*b*^	*29.47 (7.74)* ^*c*^	< 1.000 × 10^−6^	29.68 (7.75) ^a^	27.93 (7.01) ^a^	0.153
**Oxygen saturation (%), mean (SD)**	*95.75 (5.96)* ^*a*^	*94.33 (4.06)* ^*b*^	*84.46 (17.13)* ^*c*^	< 1.000 × 10^−6^	83.92 (17.58) ^a^	88.53 (12.48) ^a^	0.091
**Low eosinophil cell count (%), mean (SD)**^**e**^	*1.49 (1.72)* ^*a*^	*1.04 (1.75)* ^*b*^	1.11 (2.06) ^b^	< 1.000 × 10^−6^	1.14 (2.17) ^a^	1.06 (1.47) ^a^	0.898
**High neutrophil cell count (%), mean (SD)**^**e**^	72.93 (26.60) ^a^	*74.48 (13.60)* ^*a*^	*86.36 (45.66)* ^*b*^	< 1.000 × 10^−6^	83.36 (11.11) ^a^	103.20 (114.15) ^a^	0.953
**High lymphocyte cell count (%), mean (SD)**^**e**^	19.16 (12.76) ^a^	*18.95 (11.76)* ^*a*^	*13.62 (10.42)* ^*b*^	< 1.000 × 10^−6^	13.54 (9.86) ^a^	13.02 (8.48) ^a^	0.715
**D-Dimer (ug/L), mean (SD)**	1367.00 (1229.79) ^a^	*1330.51 (1579.57)* ^*a*^	*3157.68 (5082.00)* ^*b*^	< 1.000 × 10^−6^	3149.66 (4678.84) ^a^	3554.24 (7478.75) ^a^	0.065
**CRP (mg/L), mean (SD)**	*54.36 (81.89)* ^*a*^	*79.16 (70.98)* ^*b*^	*146.49 (102.01)* ^*c*^	< 1.000 × 10^−6^	*154.08 (104.90)* ^*a*^	*113.26 (76.33)* ^*b*^	0.013

*ACE* angiotensin converting enzyme, *BMI* body mass index, *CRP* C reactive protein, *F* female, *M* male, *n* number, *SD* standard deviation

^a, b, c^ Groups with different letters indicate significant differences in proportions or means; values for significantly different groups are shown in italics

^d^ Individuals with missing data were exclude for the percentage calculation

^e^ Lowest eosinophil count and highest lymphocyte and neutrophil counts in hospitalized patients

^f^ Test results of the comparisons between mild-to-moderate and severe groups

^g^ Test results of the comparisons between severe without asthma and severe with asthma groups

To identify the genetic profile of patients with severe COVID-19 and asthma, we divided patients with severe COVID-19 into two subgroups: 58 patients with asthma and 323 patients without asthma. Asthma was diagnosed based on patient interviews and medical history. However, no information regarding disease severity or sub phenotypes (e.g., atopy and airway hyperresponsiveness) was available.

Clinical data for all patients included individual characteristics (age, height, weight, sex) and medical history, with common medications such as systemic corticosteroids and angiotensin converting enzyme (ACE) inhibitors. Additionally, a physician conducted a physical examination to document COVID-19 symptoms (cough, headache, sore throat, ageusia, anosmia, rhinorrhea, dyspnea, fever, diarrhea, myalgia, and fatigue), asthma or respiratory conditions, comorbidities, and persistent COVID-19 symptoms. Blood cell counts (eosinophils, neutrophils, and lymphocytes), D-dimer and C-reactive protein (CRP) levels were measured.

### Whole genome sequencing

DNA extraction and whole-genome sequencing (WGS) were performed at McGill Genome Center. DNA extraction involved treating samples with a lysis buffer, followed by extraction using the CMG-1091 DNA extraction kit (Perkin Elmer on a Chemagic MSM-I instrument). DNA concentration was determined using the Quant-iTPicoGreendsDNAAssay kit (ThermoFisher Scientific, P11495). For library preparation, a 25 μl aliquot from each sample at a concentration of 16 ng/μl was used with the DNA PCR-FreePrep Tagmentation kit (Illumina, 20,041,794). Libraries quality was validated through quantitative PCR using a DNA High Sensitivity Reagent Kit (Perkin Elmer Lab Chip GX, CLS760672). Twenty-seven libraries were combined in equimolar proportions, loaded into an Illumina S4 flow cell, and sequenced on the Illumina NovaSeq 6000 [25], using the NovaSeq 6000 S4 Reagent Kit v1.5 (Illumina,20,028,312). Data from WGS were analyzed for variant detection using the GenPipesDnaSeq pipeline [26]. The reads were aligned to the human reference genome (build GRCh38) using BWA-mem aligner [27]. Then, mapping accuracy was enhanced in proximal insertion and deletion regions using GATK IndelRealigner through the GATK [28, 29] and Picard programs (http://broadinstitute.github.io/picard/). Duplicate reads were labeled using Picard Mark Duplicates and quality scores were enhanced using the GATKBaseRecalibrator. Single nucleotide variants (SNVs) were detected using GATK Haplotype Caller in GVCF mode, which enabled efficient merging of multiple samples into a single variant file downstream. Samples within each cohort were merged using GATK-combined GVCFs and genotyped using Genotype GVCFs.

Quality control measures were performed during the alignment and genotype calling phases. Samples with a mean coverage below 30x were initially enhanced through top-up procedures and contamination estimation was performed using verifyBAMid2 [30]. Concordance assessments of genotypes and sexes were conducted to address potential sample mix-up, by comparing next-generation sequencing (NGS) data and SNP array information using NGS checkmate [31] and GATK cross-check fingerprints, as necessary. Moreover, variant counts in the samples were compared. Subsequently, quality filtering was applied to both individuals and genotypes using PLINK v2.0 (www.cog-genomics.org/plink/2.0/), which was guided by data completeness levels, and aimed to eliminate individuals with high coefficients of relationship [32]. The criteria for fulfillment included: a genotype call rate > 95%, an individual call rate surpassing 90%, a Hardy–Weinberg equilibrium (HWE) *P*-value > 10^−4^, and a minor allele frequency (MAF) of at least 0.5%. Additionally, a kinship value threshold of 0.177 (KING kinship coefficients scaled to 0.5 for duplicates) was used to detect duplicate samples and first-degree relationships between samples (including parent–child and sibling–sibling pairs). In these cases, only one individual from each pair was analyzed. Following the implementation of these primary filters, 13,185,383 variants and 2131 individuals were retained for further analyses.

### Genetic analyses

Participants’ phenotypic data were compared using both group-wise analyses and comparative investigations for sex-based differences. Categorical variables were compared using the chi-square test or Fisher’s exact test, as appropriate. Continuous variables were assessed using analysis of variance (ANOVA) or Kruskal-Wallis tests, followed by Bonferroni post hoc analysis to determine specific group differences. SPSS v28.0.1 was used for the analyses and statistical significance was set at *P* < 0.05. For subsequent genome-wide association study (GWAS) analyses, covariates were selected based on test results and a comprehensive literature review to select confounding variables and avoid mediating variables. Principal components reflecting genotypic diversity among participants were computed and incorporated as covariates into the analysis models. This step aims to effectively address the population stratification.

The first logistic regression analysis was conducted to compare individuals with severe COVID-19 to those with mild-to-moderate forms. This model incorporated the first 10 principal components along with age and sex as covariates. Subsequently, a logistic regression analysis was conducted to compare individuals with severe COVID-19 and asthma to those without asthma. This model incorporated as covariates the first 10 principal components, the lowest values of eosinophil counts [16, 33–35], and the highest values of neutrophil counts of each individuals [36–38]. These counts were incorporated into the model due to the frequent association of pre-existing eosinophilia with allergic asthma in individuals with asthma, and the association of non-allergic type 2 asthma with neutrophil activation [39]. Eosinopenia and neutrophilia are recognized biomarkers for severe COVID-19. Systemic corticosteroids are also included as covariate because of their frequent usage in the management of severe asthma and for treating severe COVID-19 cases as well [18].

Both models utilized PLINK v2.0 (www.cog-genomics.org/plink/2.0/) on the Digital Research Alliance of Canada’s supercomputer (alliancecan.ca). To address convergence issues, both models employed the firth-fallback option, enabling the Firth regression when logistic regression failed to converge [32]. Moreover, continuous covariates were standardized for variance normalization. A significance threshold of 1 × 10^−6^ was considered [40].

## Results

### Clinical analyses

The study involved 2131 participants, with a mean age of 60.34 years (± 20.26) and an average body mass index (BMI) of 27.66 kg/m^2^ (± 6.50). Sex distribution was almost equal, with 49.50% females and 50.49% males. Of these participants, 80.85% (*n* = 1723) were hospitalized and 19.14% (*n* = 408) were treated as outpatients. Among the 381 patients with severe COVID-19 and known asthma status, 15% (*n* = 58) had asthma (Table 1).

Table 1 highlights the significant differences between patients with severe COVID-19 and those with mild-to-moderate disease manifestations. The average age exhibited by the severe group (63.93 ± 15.98 years) is higher than that in mild-to-moderate group (58.82 ± 20.66 years). Moreover, the average BMI in the severe group (28.89 ± 7.05 kg/m^2^) was higher than that in mild-to-moderate group (27.83 ± 6.44 kg/m^2^). The severe category was predominantly male, whereas the mild-to-moderate category had a higher number of females. When assessing immune cell types, individuals in the severe COVID-19 group had an elevated neutrophil count (86.36% ± 45.66%) and lower eosinophil (1.11% ± 2.06%) and lymphocyte (13.62% ± 10.42%) counts. They also exhibited elevated CRP (146.49 ± 102.01 mg/L) and D-dimer levels (3157.68 ± 5082.00 μg/L). Moreover, the two groups experienced dyspnea during hospitalization: 90% (*n* = 323) of patients in the severe group and 63% (*n* = 518) of patients in the mild-to-moderate group. The use of ACE inhibitors was significantly higher in the severe group (37%) than that in the mild-to-moderate group (23%).

When comparing patients with severe COVID-19 with and without asthma, we observed certain differences. Specifically, the BMI was significantly higher in patients with severe COVID-19 and asthma (33.70 ± 8.49 kg/m^2^) in comparison to those without asthma (28.17 ± 6.37 kg/m^2^). Moreover, in the severe group with asthma, CRP levels were lower (113.26 ± 76.33 mg/L) in comparison to the group without asthma (154.08 ± 104.90 mg/L).

Table 2 highlights the significant sex-based differences within the mild-to-moderate and severe groups and delineates the clinical characteristics based on sex. In the mild-to-moderate COVID-19 group, male patients had a higher hospitalization rate (74%, *n* = 379) than female patients (63%, *n* = 380). The male patients had a significantly higher average BMI (28.35 ± 6.18 kg/m^2^) in comparison to female patients (27.40 ± 6.64 kg/m^2^). Moreover, 27% of male patients in the same disease group received ACE inhibitor treatment, in contrast to 20% of female patients. The biological test results demonstrated that severe COVID-19 in male patients had higher neutrophilia (87.69% ± 44.38%) compared to mild-to-moderate cases (76.92% ± 14.67%). Similarly, male patients with mild-to-moderate COVID-19 exhibited a higher incidence of lymphopenia (16.79% ± 10.42%) and elevated CRP levels (93.32 ± 73.33 mg/L) compared to females (21.08% ± 12.61% and 64.09 ± 65.22 mg/L), respectively.

**Table 2:** Characteristics of patients with severe COVID-19 categorized by gender

	Patients with COVID-19 (*n* = 1499)				
	Mild-to-moderate (*n* = 1110)		Severe (*n* = 389)		*P* value^g^
	Male (*n* = 510)	Female (*n* = 600)	Male (*n* = 245)	Female (*n* = 144)	
**Age, mean (range)**	60.39 (0.20–98.70) ^a^	57.49 (1.70–102.70) ^a^	65.09 (29.40–94.80) ^b^	61.95 (0.70–97.70) ^a b^	0.050 × 10^−4^
**Age, median**	61.30	56.55	66.90	63.45	
**BMI (kg/m**^**2**^**), mean (SD)**	*28.35 (6.18)* ^*a*^	*27.40 (6.64)* ^*b*^	28.77 (6.88) ^a^	29.10 (7.40) ^a,b^	0.003
**Hospitalization, n (%)**	*379 (74)* ^*a*^	*380 (63)* ^*b*^	245 (100) ^c^	144 (100) ^c^	< 1.000 × 10^−6^
**Dyspnea, n (%)**^**e**^	244 (66) ^a^	274 (61) ^a^	204 (90) ^b^	119 (90) ^b^	< 1.000 × 10^−6^
**Systemic corticosteroids, n (%)**^**e**^	38 (8) ^a^	34 (6) ^a^	14 (6) ^a^	16 (11) ^a^	0.169
**ACE inhibitors, n (%)**^**e**^	*125 (27)* ^*a*^	*106 (20)* ^*b*^	94 (40) ^c^	46 (32) ^a,c^	< 1.000 × 10^−6^
**Respiratory rate (breaths per minute), mean (SD)**	23.19 (6.80) ^a^	22.09 (4.53) ^a^	29.38 (7.68) ^b^	29.63 (7.86) ^b^	< 1.000 × 10^−6^
**Oxygen saturation (%), mean (SD)**	93.95 (4.79) ^a^	94.71 (3.13) ^a^	84.14 (17.08) ^b^	85.04 (17.27) ^b^	< 1.000 × 10^−6^
**Low eosinophil cell count (%), mean (SD)**^**f**^	1.16 (2.26) ^a^	0.93 (1.04) ^a^	1.18 (2.42) ^a^	1.01 (1.24) ^a^	0.798
**High neutrophil cell count (%), mean (SD)**^**f**^	*76.92 (14.67)* ^*a*^	*72.02 (12.18)* ^*b*^	*87.69 (44.38)* ^*c*^	*84.08 (47.85)* ^*d*^	< 1.000 × 10^−6^
**High lymphocyte cell count (%), mean (SD)**^**f**^	*16.79 (10.42)* ^*a*^	*21.08 (12.61)* ^*b*^	12.76 (9.89) ^c^	15.08 (11.15) ^a,c^	< 1.000 × 10^−6^
**D-Dimer (μg/L), mean (SD)**	1389.37 (1910.60) ^a^	1265.30 (1103.31) ^a^	3529.29 (5694.89) ^b^	2553.82 (3831.71) ^b^	< 1.000 × 10^−6^
**CRP (mg/L), mean (SD)**	*93.32 (73.33)* ^*a*^	*64.09 (65.22)* ^*b*^	154.11 (100.45) ^c^	132.52 (103.79) ^c^	< 1.000 × 10^−6^

*ACE* angiotensin converting enzyme, *BMI* body mass index, *CRP* C-reactive protein, *F* female, *M* male, *n* number, *SD* standard deviation

^a, b, c, d^ Groups with the same letter indicate insignificant differences in proportions or means; values for significantly different groups are shown in italics. Only differences between sex groups of the same severity group were considered

^e^ Individuals with missing data were exclude for the percentage calculation

^f^ Lowest eosinophil count and highest lymphocyte and neutrophil counts in hospitalized patients

^g^ Test results for the analyses comparing the four groups

### Genetic analysis

**Fig. 3:**
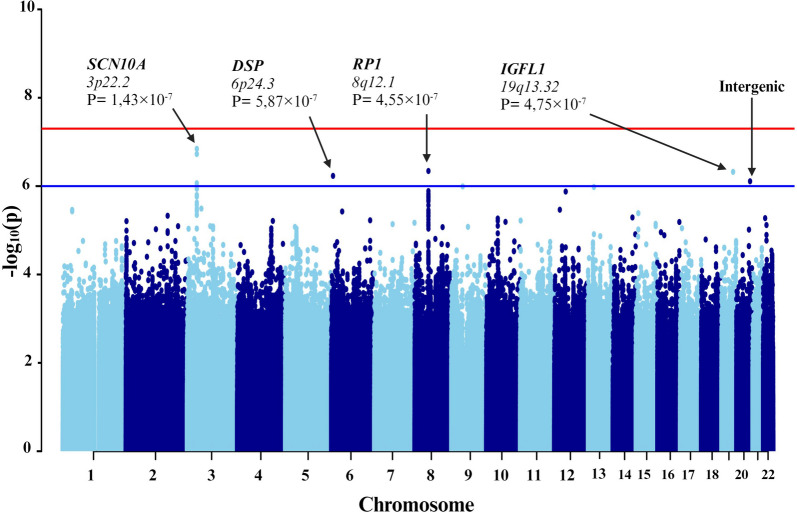
Manhattan plot of the genome-wide association study (GWAS) between mild-to-moderate and severe COVID-19. The GWAS results are shown on the y-axis as -log10 (*P*-value), and on the x-axis is the chromosomal location. The red horizontal line illustrates the genome-wide association threshold (*P* < 5 × 10^−8^) and the blue line denotes the suggestive genome-wide association threshold (*P* < 1 × 10^−6^). The Manhattan plot is generated using the qqman package in R (v4.2.1) [41]

First, individuals with mild-to-moderate COVID-19 and those with severe symptoms were compared (Fig. 3). To counteract technical biases and address population stratification, 10 principal components were included as covariates in the analyses, along with age and sex. The results indicated a significant association between seven genetic variants and severe COVID-19 (Table 3).

**Table 3:** Significant associations with a severe form of COVID-19

CHR	SNP^a^	HGVS name^b^	Effect allele	Freq^c^	Nearest gene	OR	Log (OR) SE	L95	U95	Z STAT	*P*
**3**	rs6599261	g.38793351 T > A	*A*	0.33	*SCN10A*	1.629	0.093	1.358	1.954	5.261	1.431 × 10^−7^
**3**	rs9815891	g.38791506C > T	*T*	0.32	*SCN10A*	1.626	0.093	1.354	1.952	5.211	1.878 × 10^−7^
**8**	rs5891552	g.54809591del	*TA*	0.27	*RP1*	0.577	0.109	0.466	0.714	−5.044	4.547 × 10^−7^
**19**	rs1019213	g.46225995A > G	A	0.31	3747 bp from *IGFL1* 5′ end	1.607	0.094	1.336	1.933	5.036	4.751 × 10^−7^
**6**	rs4960330	g.7573723G > T	*G*	0.49	*DSP*	1.551	0.088	1.306	1.842	4.995	5.868 × 10^−7^
**20**	rs4809972	g.54812721G > C	*C*	0.05	161,552 bp from *DOK5* 3′ end	2.717	0.202	1.828	4.039	4.941	7.774 × 10^−7^
**3**	rs62244113	g.38796247G > A	*A*	0.30	*SCN10A*	1.612	0.097	1.333	1.949	4.921	8.595 × 10^−7^

*CHR* chromosome, *Freq* frequency, *HGVS* human genome variation society, *L95* lower bound of 95% confidence interval, *lLog (OR) SE* standard error of log odds ratio (beta), *OR* odds ratio, *P P*-value, *U95* upper bound of 95% confidence interval, *Z STAT* Z statistic

^a^ SNPs identified using data from dbSNP155 from the UCSC Genome Browser (https://genome.ucsc.edu)

^b^ HGVS names and distance from gene transcription start sites calculated from the UCSC Genome Browser on Human genome build GRCh38/hg38

^c^ Frequency of the effect allele

**Fig. 4:**
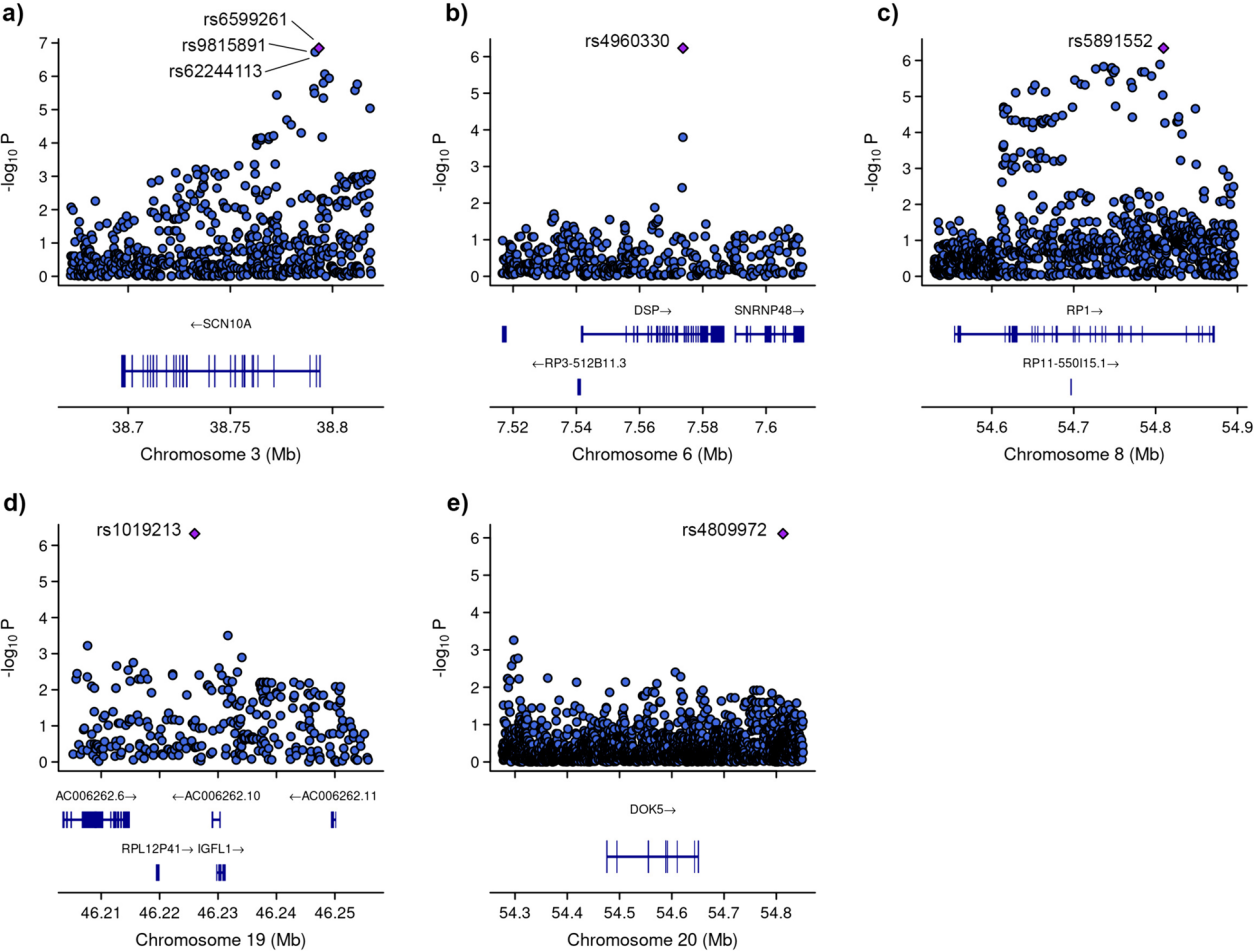
Zoom at associated loci with a severe form of COVID-19. Figure shows 25 kb regions for (**a**) sodium voltage-gated channel alpha subunit 1 (*SCN10A*), (**b**) desmoplakin (*DSP*), (**c**) RP1 axonemal microtubule-associated (*RP1*) and (**d**) IGF like family member 1 (*IGFL1*) genes as well as 200 kb region for **e)** docking protein 5 (*DOK5*) gene. The genome-wide association study (GWAS) results are shown on the y-axis as -log10 (*P*-value), and on the x-axis is the chromosomal location in Mb. At the bottom of each are the genes found in corresponding locus according to Ensemble Database library for homo sapiens v86. The plots are generated using the locuszoomr package in R (v4.3.0)

Among these variants, three (rs6599261, rs9815891, and rs62244113) were located within sodium voltage-gated channel alpha subunit 1 (*SCN10A*) at locus *3p22.2*, with *P*-values ranging from 8.595 × 10^−7^ to 1.431 × 10^−7^ (Fig. 4). Another variant was located within the RP1 axonemal microtubule-associated (*RP1*) at locus *8q12.1* (*P*-value = 4.547 × 10^−7^). Additionally, rs1019213 is positioned 3747 base pairs (bp) upstream of IGF like family member 1 (*IGFL1*) at locus *19q13.32*. Another intergenic variant, rs4809972, was positioned 161,552 bp downstream of docking protein 5 (*DOK5*) at locus *20q13.2* (*P*-value = 7.774 × 10^−7^). Within desmoplakin (*DSP*) located at locus *6p24.3*, the variant rs4960330 was identified (*P*-value =5.868 × 10^−7^).

**Fig. 5:**
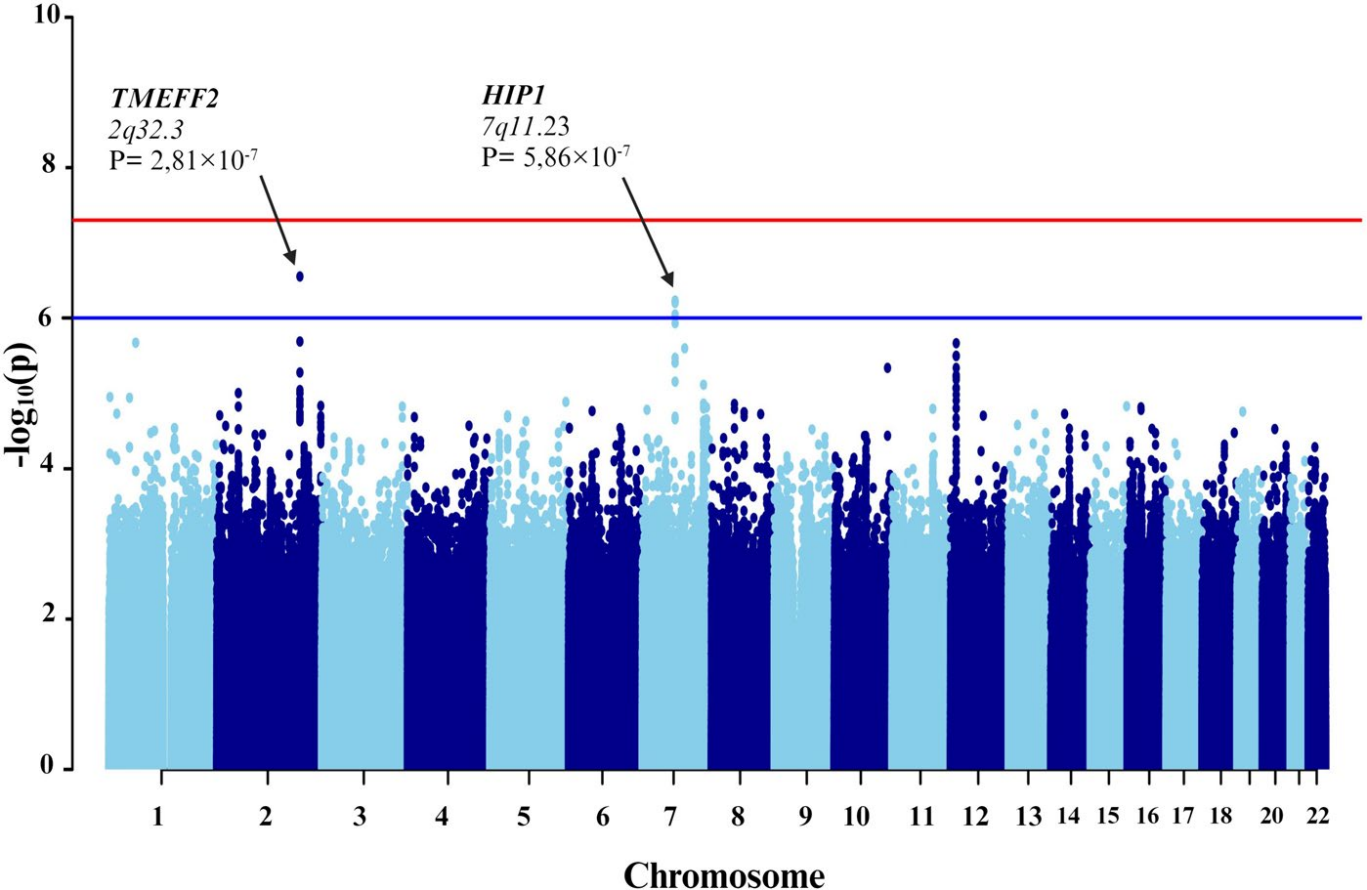
Manhattan plot of the genome-wide association study (GWAS) between patients with severe COVID-19 plus asthma and those without asthma. The GWAS results are shown on the y-axis as -log10 (*P*-value), and on the x-axis is the chromosomal location. The red horizontal line illustrates the genome-wide association threshold (*P* < 5 × 10^−8^) and the blue line indicates the suggestive genome-wide association threshold (*P* <  1 × 10^−6^). The Manhattan plot is generated using the qqman package in R (v4.2.1) [41]

**Fig. 6:**
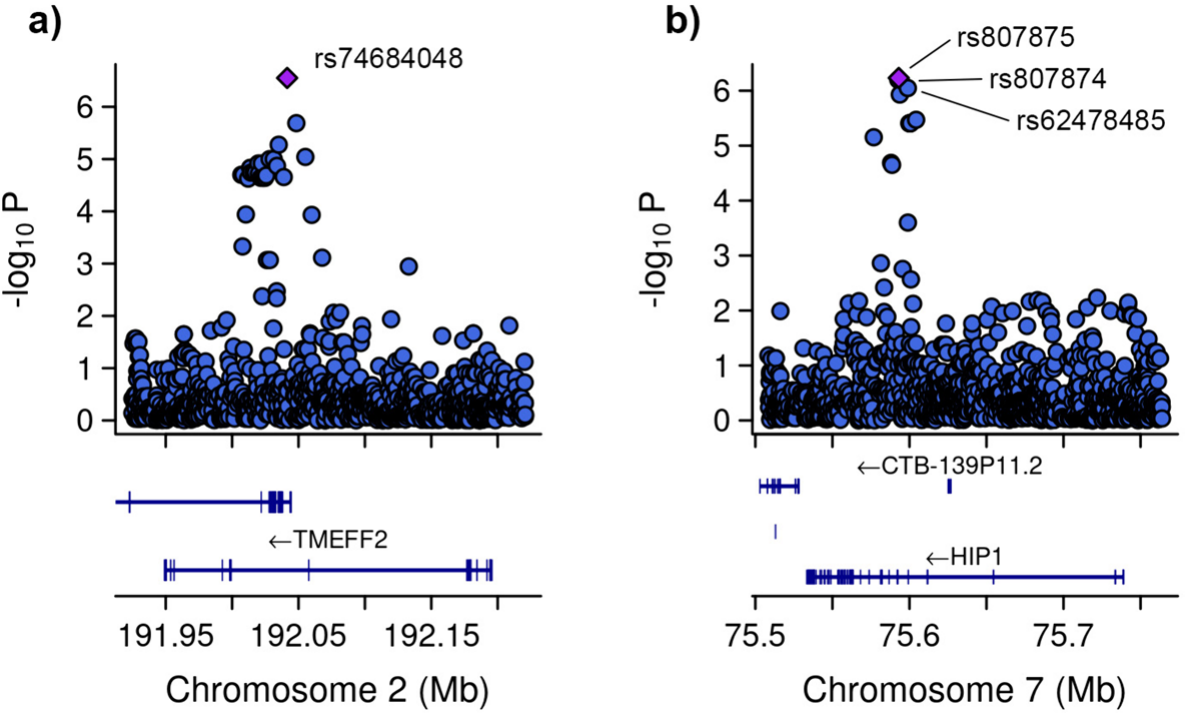
Zoom at associated loci with a severe form of COVID-19 with asthma. Figure shows 25 kb regions for (**a**) transmembrane protein with EGF like and two follistatin like domains 2 (*TMEFF2*) and (**b**) huntingtin interacting protein 1 (*HIP1*) genes. The genome-wide association study (GWAS) results are shown on the y-axis as -log10 (*P*-value), and on the x-axis is the chromosomal location in Mb. At the bottom of each are the genes found in corresponding locus according to Ensemble Database library for homo sapiens v86. The plots are generated using the locuszoomr package in R (v4.3.0)

Subsequent GWAS was conducted between the groups with severe COVID-19 and asthma and those without asthma (Fig. 5). In addition to the 10 principal components, additional covariates included the lowest eosinophil count, highest neutrophil count, and systemic corticosteroid medication. Four genetic variants were associated. Specifically, one of the variant rs74684048 was located within the transmembrane protein with EGF like and two follistatin like domains 2 (*TMEFF2*) at locus *2q32.3* (*P*-value = 2.807 × 10^−7^). Three additional variants (rs807875, rs807874, and rs62478485) were detected within the huntingtin interacting protein 1 (*HIP1*) at locus *7q11.23*, with *P*-values ranging from 8.953 × 10^−7^ to 5.860 × 10^−7^ (Table 4 and Fig. 6).

**Table 4:** Significant associations with a severe form of COVID-19 with asthma

CHR	SNP^a^	HGVS name^b^	Effect allele	Freq^c^	Nearest gene	OR	Log (OR) SE	L95	U95	Z STAT	P
**2**	rs74684048	g.192041442A > G	*G*	0.08	*TMEFF2*	11.137	0.469	4.439	27.941	5.136	2.807 × 10^−7^
**7**	rs807875	g.75593054A > G	*G*	0.83	*HIP1*	5.148	0.328	2.707	9.790	4.996	5.860 × 10^−7^
**7**	rs807874	g.75592752C > T	*T*	0.83	*HIP1*	5.163	0.330	2.706	9.849	4.981	6.315 × 10^−7^
**7**	rs62478485	g.75598931A > G	*G*	0.18	*HIP1*	4.957	0.326	2.618	9.389	4.913	8.953 × 10^−7^

*CHR* chromosome, *Freq* frequency, *HGVS* Human Genome Variation Society, *L95* lower bound of 95% confidence interval, *lLog (OR) SE* standard error of log odds ratio (beta), *OR* odds ratio, *P P*-value, *U95* upper bound of 95% confidence interval, *Z STAT* Z statistic

^a^ SNPs were identified using data from dbSNP155 from the UCSC Genome Browser (https:// genome. Ucsc. edu)

^b^ HGVS names and distances from gene transcription start sites calculated from the UCSC Genome Browser on Human genome buildGRCh38/hg38

^c^ Frequency of the effect allele

## Discussion

The primary objective of this study was to conduct a comprehensive pan-genomic analysis of individuals from BQC19, a representative sample of the Quebec population. The study aimed to acquire deeper insights into the genetic and clinical aspects of severe COVID-19 with and without asthma comorbidity. To reach this goal, it is one of the very few studies to compare genomes of patients with mild-to-moderate COVID-19 to the ones with severe COVID-19, allowing to better document the genomic profile specific to the severe form of COVID-19.

Two distinct genetic profiles were identified: one for individuals with severe COVID-19, and another for those with severe COVID-19 alongside asthma. The robust findings of this study were supported by the representative population, rendering results potentially applicable to the Quebec population. This analysis revealed multiple genomic loci associated with COVID-19 severity with or without asthma comorbidity, including *DSP*, *HIP1* and *RP1* genes. These genes have been associated through genomic or proteomic analyses in previous studies [42–44].

Precise asthma phenotyping (based on the Global Initiative for Asthma, 2023) [45] could distinguish the protective effect of allergic asthma from the potential risk associated with severe asthma in severe COVID-19 cases. Moreover, the genetic profiles identified in this study did not encompass new COVID-19 variants, as the samples were collected and analyzed prior to this emergence. Although these findings are significant, assessing them in an independent cohort is fundamental to enhance the validity of results. Additionally, increasing the sample size of patients with asthma could enhance the statistical robustness of the study, enabling a precise analysis.

When comparing mild-to-moderate and severe COVID-19 groups, seven significant variants were identified. The most prominent signal was observed at locus *3p22.2*, in which three specific variants (rs6599261, rs9815891, and rs62244113) were identified. *SCN10A* codes for the voltage-gated sodium channel Nav1.8. Shiers et al. showed that the ACE2 receptor, responsible for SARS-CoV-2 viral entry into host cells, is predominantly expressed in neuronal nociceptors labeled by this sodium channel [46]. This implies a potential route for the infection of nociceptors through the respiratory airways due to *ACE2* expression. An elevated *ACE2* expression was observed in the thoracic dorsal root ganglia, which house nociceptors responsible for lung innervation [47, 48]. This is significant because of lungs being a prime site for SARS-CoV-2 viral replication [49]. The phenotypic data complement this observation with the elevated use of ACE inhibitors in severe COVID-19 cases. This could be attributed to the potential of ACE inhibitors to increase ACE2 receptor expression, enhancing viral entry [50]. Recent studies suggested an association between *SCN10A* and chronic obstructive pulmonary disease (COPD) [51]. Dyspnea prominently characterizes COPD, which aligns with previous clinical observations. *SCN10A* is also associated with cardiovascular diseases. Previous GWAS highlighted the significance of genetic variations in *SCN10A* on cardiac conduction [52], a factor associated with unanticipated cardiac arrest [53]. This trait is associated with a higher susceptibility to COVID-19 [54–56]. Moreover, research has established a correlation between cardiac conduction aberrations and SARS-CoV-2-related complications. The systemic inflammatory response to COVID-19, referred to as the “cytokine storm”, can adversely affect cardiac function and disrupt cardiac conduction [57, 58]. The significance of *SCN10A* has thus been assessed in both pulmonary and cardiac disorders, in accordance with recognized risk factors for severe COVID-19.

A new variant was identified at locus *8q12.1* in the *RP1* gene. A recent study highlighted the significance of *RP1* in association with SARS-CoV-2 and Middle East respiratory syndrome (MERS) viruses, indicating its role in facilitating viral infections and severe disease complications [42]. However, its precise contribution to disease pathophysiology remains uncertain.

The variant rs4960330, at locus *6p24.3* within *DSP*, is associated with severe COVID-19. A recent investigation demonstrated elevated DSP levels in acute COVID-19 cases [43]. Another study revealed 23 *DSP* variants associated with idiopathic pulmonary fibrosis (IPF). Among these, rs2076295 and rs2744371, were associated with increasing *DSP* expression in the respiratory epithelium of IPF-affected lungs [59, 60]. Recent findings indicate that up to 11% of patients develop IPF [61] after recovery from COVID-19 acute phase. rs2076295 is also associated with interstitial lung abnormalities [60], a condition frequently observed in patients with COPD [62].

The variant rs1019213 at locus *19q13.32,* is positioned 3747 bp upstream of *IGFL1*. Elevated *IGFL1* expression is associated with poor prognosis in lung adenocarcinoma [63]. However, the correlation between COVID-19 and lung cancer remains uncertain.

We identified the final intergenic variant rs4809972 at locus *20q13.2*, which was 161,552 bp downstream of *DOK5*. Another variant near the *DOK5* gene, rs60684837, was previously associated with COVID-19 mortality in the western Indian population [64]. This gene is also associated with obesity [65] and diabetes, [66] two comorbidities recurrently identified as COVID-19 risk factors in numerous investigations [67]. Additionally, another study indicated that overexpression of *DOK5* in fibroblasts contributes to the progression of IPF [68]. However, it is important to interpret these results with caution due to the distance between the variant and the nearest gene.

The second GWAS compared the genetic profiles of individuals with severe COVID-19 and asthma to those without asthma. Correlation between COVID-19 and asthma is an ongoing research subject. The relationship between asthma and COVID-19 varies across asthma phenotypes. For instance, allergic asthma appears to offer protection through the IL-13 pathway [69], whereas severe asthma appears to be associated with severe COVID-19 outcomes through the ACE2 receptor pathway [70]. Genomic investigations can help elucidate the biological nature of these relationships. This study identified two genomic regions containing four variants significantly associated with the combined phenotypes of severe COVID-19 and asthma.

The variant rs74684048, located at locus *2q32.3* within *TMEFF2*, is associated with a specific phenotype. *TMEFF2* was genetically associated with submucosal eosinophils in bronchial brushing samples of patients with severe asthma [71]. An epigenome-wide association study revealed an association between DNA methylation of *TMEFF2* and lung function [72]. Additionally, other studies indicated that methylation in the *TMEFF2* promoter regions reduces its activity, potentially contributing to lung tumor development [73]. There is no direct association between *TMEFF2* and COVID-19. Further research is required to understand the function of this gene in both asthma and COVID-19.

Three additional variants (rs807875, rs807874, and rs62478485) within *HIP1* at locus *7q11.23* were identified. This finding corroborates results of a study by Pairo et al., which associated *HIP1* with severe COVID-19 [44]. It is possible that *HIP1* is involved in the endocytosis process of SARS-CoV-2, as the virus enters host cells through clathrin-induced endocytosis [74], a pathway involving *HIP1* [75, 76]. Additionally, other studies have revealed elevated *HIP1* expression in lung cancer, with *HIP1* identified as a novel fusion partner of anaplastic lymphoma kinase [77, 78]. This indicates that *HIP1* may be implicated in COVID-19 through its interaction with clathrin. However, there is no distinct association between *HIP1* and asthma.

## Conclusions

This study enhances our understanding of the risk factors for severe COVID-19 and highlights the significant role of genetics in determining susceptibility to this form of the disease. It delineates a specific genetic profile of severe COVID-19 compared to mild-to-moderate form and severe COVID-19 with asthma compared to severe COVID-19 without asthma. These findings have the potential to enhance preventive strategies in patients with severe COVID-19. By combining the GWAS data from this study with forthcoming data, a potential polygenetic risk score can be developed to identify individuals with a high risk of developing severe COVID-19 in relation with their asthma status. Further investigations with precise asthma phenotyping are needed to refine and fortify these findings.

## Data Availability

All clinical and genetic data used and results generated are available from the BQC19 via data request at https://www.bqc19.ca/en/access-data.

## Abbreviations

*ABO*: ABO, Alpha 1-3-N-acetylgalactosaminyltransferase and alpha 1-3-galactosyltransferase
ACE: Angiotensin converting enzyme
ANOVA: Analysis of variance
BMI: Body mass index
BQC19: *Biobanque québécoise de la COVID-19*
*CRP*: C-reactive protein
COPD: Chronic obstructive pulmonary disease
COVID-19: Coronavirus disease 2019
*DOK5*: Docking protein 5
*DSP*: Desmoplakin
GWAS: Genome-wide association study
*HIP1*: Huntingtin interacting protein 1
HWE: Hardy-Weinberg equilibrium
*IGFL1*: IGF like family member 1
IL: Interleukin
IPF: Idiopathic pulmonary fibrosis
MAF: Minor allele frequency
MERS: Middle East respiratory syndrome
NGS: Next-generation sequencing
OAS: Oligoadenylate synthetase
RNA: Ribonucleic acid
*RP1*: RP1 axonemal microtubule-associated
RT-PCR: Reverse transcriptase polymerase chain reaction
SARS-CoV-2: Severe acute respiratory syndrome coronavirus 2
*SCN10A*: Sodium voltage-gated channel alpha subunit 1
SNV: Single nucleotide variant
Th2: Type 2 helper T cell
*TMEFF2*: Transmembrane protein with EGF like and two follistatin like domains 2
WGS: Whole-genome sequencing
WHO: World health organization
